# Predictive metabolomics of multiple Atacama plant species unveils a core set of generic metabolites for extreme climate resilience

**DOI:** 10.1111/nph.18095

**Published:** 2022-04-05

**Authors:** Thomas Dussarrat, Sylvain Prigent, Claudio Latorre, Stéphane Bernillon, Amélie Flandin, Francisca P. Díaz, Cédric Cassan, Pierre Van Delft, Daniel Jacob, Kranthi Varala, Jérôme Joubes, Yves Gibon, Dominique Rolin, Rodrigo A. Gutiérrez, Pierre Pétriacq

**Affiliations:** ^1^ Departamento de Genética Molecular y Microbiología Pontificia Universidad Católica de Chile FONDAP Center for Genome Regulation and Millenium Institute for Integrative Biology (iBio) Av Libertador Bernardo O'Higgins 340 Santiago Chile; ^2^ Univ. Bordeaux INRAE UMR1332 BFP, 33882 Villenave d'Ornon France; ^3^ Bordeaux Metabolome MetaboHUB PHENOME‐EMPHASIS 33140 Villenave d'Ornon France; ^4^ Departamento de Ecología Pontificia Universidad Católica de Chile Av Libertador Bernardo O'Higgins 340 Santiago Chile; ^5^ Institute of Ecology and Biodiversity (IEB) Las Palmeras 3425 Ñuñoa Santiago Chile; ^6^ Laboratoire de Biogenèse Membranaire, CNRS Univ. Bordeaux, UMR 5200 Villenave d'Ornon France; ^7^ 311308 Department of Horticulture and Landscape Architecture Purdue University West Lafayette IN 47907 USA; ^8^ Center for Plant Biology Purdue University West Lafayette IN 47907 USA

**Keywords:** adaptation, extreme environments, multiple species, plant metabolism, predictive metabolomics

## Abstract

- Current crop yield of the best ideotypes is stagnating and threatened by climate change. In this scenario, understanding wild plant adaptations in extreme ecosystems offers an opportunity to learn about new mechanisms for resilience. Previous studies have shown species specificity for metabolites involved in plant adaptation to harsh environments.
- Here, we combined multispecies ecological metabolomics and machine learning‐based generalized linear model predictions to link the metabolome to the plant environment in a set of 24 species belonging to 14 families growing along an altitudinal gradient in the Atacama Desert.
- Thirty‐nine common compounds predicted the plant environment with 79% accuracy, thus establishing the plant metabolome as an excellent integrative predictor of environmental fluctuations. These metabolites were independent of the species and validated both statistically and biologically using an independent dataset from a different sampling year. Thereafter, using multiblock predictive regressions, metabolites were linked to climatic and edaphic stressors such as freezing temperature, water deficit and high solar irradiance.
- These findings indicate that plants from different evolutionary trajectories use a generic metabolic toolkit to face extreme environments. These core metabolites, also present in agronomic species, provide a unique metabolic goldmine for improving crop performances under abiotic pressure.

## Introduction

Humans domesticated plants 10 000 yr ago in the hostile environments of the Fertile Crescent (Dai *et al*., 2012; Riehl *et al*., 2012). Over the years, selected crops have been improved by a variety of methods. However, current yields of domesticated therophytes are stagnating and threatened by climate change despite significant efforts to develop abiotic stress tolerance for the best ideotypes (Long *et al*., 2015). Wild plants naturally evolved mechanisms to meet abiotic constraints in natural habitats from which they cannot escape (Fatima *et al*., 2020; Signori‐Müller *et al*., 2021). In this scenario, returning to wild plant species that live and thrive in some of the harshest environments on Earth offers an opportunity to find new strategies for crop improvement (Castañeda‐Álvarez *et al*., 2016). Recent studies have pinpointed relevant metabolic clusters in adaptation to extreme environments in plants harvested in high mountains, deserts and salt lands (Dussarrat *et al*., 2021). These adaptive mechanisms involved the accumulation of amino acids (Lugan *et al*., 2010) as precursors of secondary metabolites, and carotenoids (Cui *et al*., 2019) and polyphenols (Hashim *et al*., 2020) as processors of reactive oxygen species (ROS). In addition, most of these studies were carried out on a unique or limited number of species (Dussarrat *et al*., 2021), which, combined with high biochemical diversity, led to highly specific metabolic markers involved in adaptive mechanisms exclusive to the species or environment (Peters *et al*., 2018; Dussarrat *et al*., 2021).

The metabolome is an excellent integrative system to predict plant environment because it carries imprints of omic inferences and environmental influences (Kosmacz *et al*., 2020; Lewis & Kemp, 2021). Ecological metabolomics aims to study the environmental impact on metabolic responses, acclimation and adaptation processes in natural ecosystems. Applying untargeted ecological metabolomics on multiple species could unravel universal plant adaptive strategies to abiotic factors in their natural environment (Poorter *et al*., 2012; Umair *et al*., 2019; Sardans *et al*., 2020; Wong *et al*., 2020). However, this approach has focused primarily on analysis of phytochemical diversity. Plant metabolomes were recently used to predict phenotypic traits such as yield and stress resistance (Zhu *et al*., 2018; Luna *et al*., 2020; Szymański *et al*., 2020) within specific species. By exploiting multiple species, previous studies reported strong relationships between growth rate and biomass composition (Roch *et al*., 2020) and between phytochemical diversity and environmental conditions of plants growing in alpine regions (Defossez *et al*., 2021). Interestingly, several phenotypic traits predicted from plant metabolism were further used to predict complex output such as plant fitness but remain tedious to collect (Laughlin *et al*., 2012; Laughlin & Messier, 2015). Thus, ecological metabolomics could be used to uncover readily measurable soft traits that can predict complex outputs such as plant fitness. Adaptation to extreme environments is thought to rely on specialized secondary metabolic pathways often considered to be species‐specific (Moghe & Last, 2015; Scossa & Fernie, 2020). However, generic mechanisms may also exist. To test this hypothesis, large‐scale metabolomics in multiple wild species are needed to unveil general metabolic interactions with environmental factors and propose adaptive roles for specific metabolites (Wong *et al*., 2020).

The Atacama Desert is the driest nonpolar desert on Earth. In addition to extreme aridity, the Atacama is characterized by high solar radiation, extreme daily temperature oscillations, high soil salinity and low nitrogen content (Eshel *et al*., 2021). Although multiple abiotic factors are intense enough to severely limit plant life, this desert hosts tens of plant species (Jordan & Kirk‐Lawlor, 2014; Díaz *et al*., 2016, 2019), thus bestowing a unique opportunity to analyse adaptive metabolic plant responses to abiotic stress in an entire ecosystem. The present study aimed to characterize the metabolic profiles of 24 dominant plant species in 19 different sites along an altitudinal transect in the Atacama Desert. Biological and environmental diversity was used to question the extent to which adaptation to extreme environments relies on generic metabolic mechanisms.

To meet these ambitious objectives, multiplatform metabolomics covering primary compounds including carbohydrates, amino and organic acids, fatty acids and secondary metabolites revealed metabolic features that participate in environmental adaptation. Subsequent machine learning modelling of this comprehensive dataset via a generalized multilinear‐based statistical approach established that the metabolome of these 24 extremophile plants was an excellent integrative predictor of plant environments. Moreover, our analysis uncovered a common set of metabolites associated with extreme climate resilience.

## Materials and Methods

### Plant materials and sampling

**Fig. 1:**
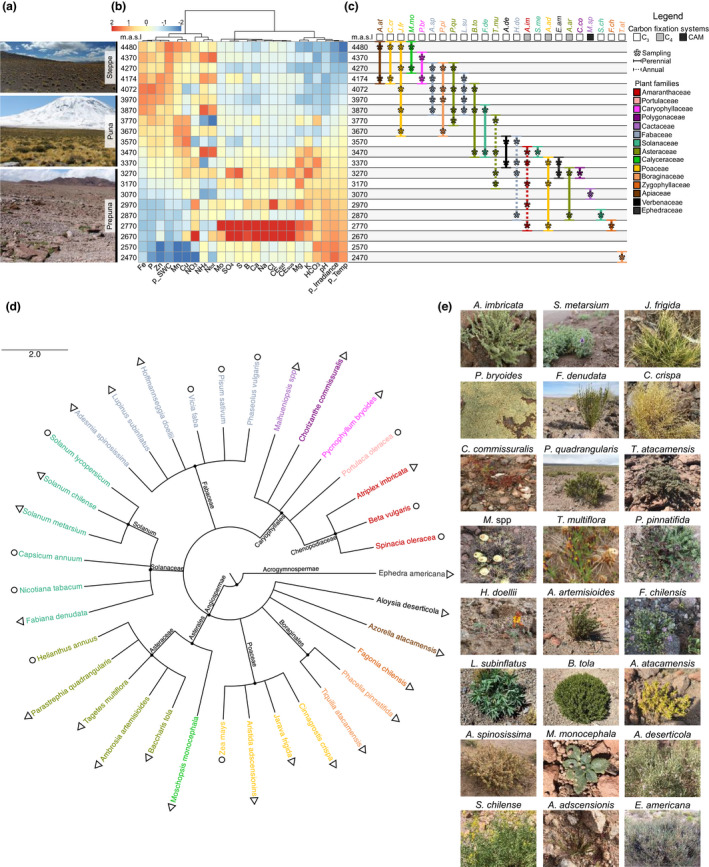
Depiction of Atacama plant diversity despite extreme conditions. (a) Picture of the three vegetation belts. (b) Description of the environmental conditions observed along the elevation gradient (Pearson correlation, *P* < 0.05). CE, electrical conductivity; Ntot, total nitrogen; SWC, soil water content; Temp, temperature; p_ represents a partially predicted parameter. (c) Description of the sampling site ranges and main characteristics (carbon fixation systems or lifespan) of the collected plant species. (d) Analysis of the taxonomic relationships between Atacama species and between Atacama and agronomic or ornamental plant species. Triangles represent the Atacama plants while circles represent the agronomic and ornamental species. (e) Pictures of the Atacama plant species collected.

The aerial parts of 24 plant species belonging to 14 plant families (Supporting Information Table S1) were collected in their natural conditions from the Chilean Atacama Desert (Talabre‐Leja transect (Díaz *et al*., 2016), 22–24°S). For each species, a minimum of three biological replicates composed of multiple plants was collected. Each species was collected at one to six distinct elevation levels (Fig. 1) depending on biological availability, directly snap frozen in liquid nitrogen, brought back to the laboratory on dry ice and stored at −80°C until freeze‐drying. Sampling was performed during two consecutive days (6–7 April 2019) between 09:30 and 17:30 h. The time variation between samplings in different environments did not impact starch content, suggesting stable central metabolism during the sampling period (Fig. S1). Additionally, crops and ornamental plant species including *Capsicum annuum*, *Phaseolus vulgaris*, *Spinacia oleracea*, *Vicia faba*, *Pisum sativum*, *Beta vulgaris*, *Portulaca oleracea*, *Helianthus annuus*, *Zea mays*, *Nicotiana tabacum* and *Solanum lycopersicum* were grown in multiple natural conditions in France. The aerial parts of those plants were harvested, snap‐frozen in liquid nitrogen and stored at −80°C until freeze‐drying. All freeze‐dried material from extremophiles and common plants was kept at −80°C until further analysis.

### Environmental data

Climatic conditions were characterized using two meteorological stations (at 3060 and 4090 m above sea level (m asl)), which measured temperature, humidity and solar irradiance as well as precipitation or soil moisture levels every hour throughout 2018–2019 (Eshel *et al*., 2021). In addition, soil chemical properties including pH and contents of nitrate, ammonium, Olsen phosphorous, zinc, potassium, manganese, copper, iron, boron, molybdenum, sulphur, calcium, manganese, sodium, chlorine, bicarbonate salt and silt were measured and described for over 3 yr (Eshel *et al*., 2021).

### Metabolite extraction

Using 20 mg of lyophilized plant material (from crops, ornamental and Atacama plants), robotized extractions of metabolites were performed according to an ethanol fractionation protocol (Luna *et al*., 2020), which targets a wide range of semipolar plant biochemicals including primary compounds (soluble sugars and starch, organic and amino acids, total proteins) and specialized metabolites (terpenes, phenolics, alkaloids). In parallel, 10 mg of lyophilized plant samples were used to extract fatty acyls from total lipids as described previously (Domergue *et al*., 2010).

### Metabolomics

Ethanol extracts were screened for multiple compounds (Chl, glucose, fructose, sucrose, malate, free amino acids, nitrate, total proteins and starch) based on coupled enzyme assays (Luna *et al*., 2020). The same extracts were also subjected to untargeted metabolic profiling by UHPLC‐LTQ‐Orbitrap MS (liquid chromatography‐mass spectrometry (LCMS)) using an Ultimate 3000 ultra‐high‐pressure liquid chromatography (UHPLC) system coupled to an LTQ‐Orbitrap Elite mass spectrometer interfaced with an electrospray ionization (ESI) source (ThermoScientific, Bremen, Germany) operating in both negative and positive ion modes as described previously (Luna *et al*., 2020). Separation was performed using a C18 column (C18‐Gemini, 2.0 × 150 mm, 3 µm, 110 Å; Phenomenex, Torrance, CA, USA). Full scan high‐resolution MS spectra were acquired at 240k resolution power at *m*/*z* = 200 Da. In addition, LCMS/MS acquisitions were acquired in higher‐energy collisional dissociation (HCD) mode at a normalized collision energy of 60% and 35% (ESI− and + respectively). Fatty acyls were analysed using gas chromatography coupled to a flame ionization detector (GCFID) or a mass spectrometric detector (GCMS) as detailed previously (Domergue *et al*., 2010). Biochemical phenotyping and LCMS experiments were performed for all plants (i.e. Atacama plants from 2014 and 2019, crops and ornamental plants), while GCFID and GCMS experiments were performed for 2019 Atacama plants and crops and ornamental plants exclusively.

### Processing of metabolomic data

Raw LCMS data were processed via Xcms (v.4.2) in R (v.3.6.1) (Smith *et al*., 2006) using in‐house optimized parameters(Luna *et al*., 2020) yielding 8750 detected RT–*m/z* pairs for 5130 ESI− and for 3620 ESI+ modes. Subsequent data cleaning (blank check, Δ_RT_ < 60 s, Δ*_m/z_* < 0.025 Da, coefficient of variation in quality controls < 30%) generated 4540 metabolic variables (2564 ESI− and 1976 ESI+) that were retained for chemometric analyses. Both untargeted and targeted metabolomics data were first normalized by median normalization, cube‐root transformation and Pareto scaling using MetaboAnalyst v.3 (Xia *et al*., 2015) before applying multivariate and univariate statistical analyses. The nonnormalized dataset obtained after preprocessing is available in Table S2 and deposited online (see ‘Data availability’ section).

### Generalized multilinear models

Generalised linear modelling (GLM) was performed to determine the quantitative correlation between metabolism and elevation levels used as a proxy of the plant environment. All metabolic variables that could not be measured based on detection limitations were inputted as 0 in the data matrix. The linear models were generated using the glmnet package (Friedman *et al*., 2010) in the R software (R Core Team, 2020) (v.3.6.1). Three model types were constructed (lasso, elastic net and ridge) by varying the penalty value of the elastic net as a proxy to modulate the number of variables used by the models. Thousand values ranging from 0 to 1 were tested. Internal cross‐validation was performed for construction of the models to mitigate the overfitting. The best model was chosen based on the mean square error (MSE), and the most parsimonious model within one SE of the minimal MSE was selected to perform predictions. The datasets were divided into three parts: 70% of the plants in a ‘training’ set, 20% in a ‘test’ set and 10% in a ‘validation’ set to perform real predictions using the best model developed with both training and testing sets. Stratified sampling was used to perform a uniform sampling of the individuals based on the measured elevation levels. Due to this random partitioning, 500 different simulations were performed to sample the solution space of possible predictions. In addition, 500 sets of randomly assigned elevation levels were created to test the likelihood of spurious predictions. Student tests were performed to compare the 500 results from models performed with permutated and real elevation levels. The occurrences of the metabolic variables among the 500 simulations were analysed to extract the best predictors (Table 1).

**Table 1:** Annotation of the 39 best metabolic markers.

Variable ID	Occurrence in the model	Correlation	Observation	Detected *m*/*z*	Detected RT	*P* value FDR	Ion type	Predicted *m/z*	Δ *m/z* (ppm)	Putative formula	MSI level	Putative compound
Starch	98.6	Negative	–	–	–	< 1.64E‐15	–	–	–	–	‐	–
n_2561	90.0	Negative	Mg(C_2_H_2_O_4_)*_n_* + HCOO^−^	386.93908	0.9	< 1.64E‐15	–	–	–	Mg(C_2_H_2_O4)*_n_* + HCOO^−^	–	Mg(C_2_H_2_O_4_)*_n_* + HCOO^−^
p_1777	87.4	Negative	Na(HCO_2_)*_n_* + HCOO^−^	274.87218	1.1	< 1.64E‐15	–	–	–	Na(HCO_2_)*_n_* + HCOO^−^	–	Na(HCO_2_)*_n_* + HCOO^−^
n_0601	86.6	Negative	–	233.10296	3.9	< 1.64E‐15	[M‐H]^−^	234.11024	0	C_10_H_18_O_6_	MSI3	3,6‐Dihydroxy‐2,7‐dimethyloctanedioic acid
p_2029	85.7	Negative	–	298.09560	3.6	1.03E‐12	[M+H]^+^	297.08780	4.8	C_11_H_15_N5O_3_S	MSI2	5′‐Methylthioadenosine
n_0615	84.8	Negative	–	236.05628	5.6	< 1.64E‐15	[M‐H]^−^	237.06370	0.4	C_11_H_11_NO_5_	MSI3	*N*‐Benzoylaspartic acid
p_2329	83.7	Positive	–	323.07473	7.8	8.91E‐08	[M+H]^+^	322.06890	4.9	C_15_H_14_O_8_	MSI3	Leucodelphinidin
p_0179	82.7	Negative	Fragment of 251.16	116.05708	11.5	1.64E‐15	[M+H]^+^	250.15640	4.2	C_15_H_22_O_3_	MSI3	Xanthoxin
Qt027	82.5	Positive	–	–	–	1.01E‐10	–	–	–	–	–	ND
p_3344	82.0	Negative	–	584.27182	9.1	< 1.64E‐15	[M+H]^+^	583.26820	5.9	C_34_H_37_N_3_O_6_	MSI2	*N*1,*N*5,*N*10‐Tricoumaroyl spermidine
n_3183	81.4	Negative	–	436.22628	5.4	< 1.64E‐15	[M‐H]^−^	437.02315	0.8	C_25_H_31_O_4_N_3_	MSI2	*N*1,*N*10‐Dicoumaroylspermidine
n_2074	79.3	Negative	–	349.15000	7.3	< 1.64E‐15	[M‐H]^−^	350.15727	0.4	C_15_H_26_O_9_	MSI3	Azelaic acid glycoside
n_4005	78.2	Positive	–	503.16151	1.4	1.10E‐05	[M‐H]^−^	504.16900	0.4	C_18_H_32_O_16_	MSI2	Raffinose
p_0421	79.2	Negative	–	144.10132	1.3	< 1.64E‐15	[M+H]^+^	143.09460	3.7	C_7_H_13_O_2_N	MSI2	Proline betaine
n_2571	78.3	Negative	–	387.16572	5.2	1.64E‐15	[M‐H]^−^	388.17330	1.3	C_18_H_28_O_9_	MSI2	7‐Epi‐12‐hydroxyjasmonic acid glucoside
n_4749	77.2	Negative	M: 625.14020, ESI relative: p_3426	627.14616	6.0	< 1.64E‐15	[M‐H]^−^	626.14830	1.3	C_27_H_30_O_17_	MSI3	3,3′,4′,5,7,8‐Hexahydroxyflavone; 7‐*O*‐α‐l‐rhamnopyranoside, 8‐*O*‐β‐d‐glucopyranoside
n_1843	77.1	Negative	M: 331.04562	332.04902	8.9	< 1.64E‐15	[M‐H]^−^	332.05320	0.9	C_16_H_12_O_8_	MSI3	Quercetagetin methyl ether
p_0586	73.6	Negative	–	161.09152	1.1	< 1.64E‐15	[M+H]^+^	161.09207	3.9	C_3_H_7_NO_2_	MSI2	d‐Alanyl‐d‐alanine
p_0184	73.0	Negative	M: 116.06969	117.07322	1.3	6.79E‐05	[M+H]^+^	115.06330	0.5	C_5_H_9_O_2_N	MSI2	Proline
p_2208	71.1	Positive	–	315.04863	11.1	0.000000151	[M+H]^+^	314.04270	3.8	C_16_H_10_O_7_	MSI3	Wedelolactone
n_2574	70.2	Negative	–	387.20213	5.2	1.96E‐14	[M‐H]^−^	388.20970	0.9	C_19_H_32_O_8_	MSI3	9,13‐Dihydroxy‐4‐megastigmen‐3‐one 9‐glucoside
n_5122	69.4	Negative	–	925.47889	10.0	< 1.64E‐15	[M‐H]^−^	926.48750	1.1	C_47_H_74_O_18_	MSI3	Araloside A
n_4791	69.0	Negative	–	639.13506	8.1	1.09E‐08	[M‐H]^−^	640.14280	0	C_31_H_28_O_15_	MSI3	Quercetin 3‐(6′‐ferulylglucoside)
n_0657	68.3	Positive	–	241.07186	5.0	8.10E‐06	ND	ND	ND	ND	ND	ND
n_2605	67.6	Negative	–	389.21773	8.4	< 1.64E‐15	[M‐H]^−^	390.22540	0.4	C_19_H_34_O_8_	MSI3	(3*S*,5*R*,6*S*,7*E*,9*x*)‐7‐Megastigmene‐3,6,9‐triol 9‐glucoside
n_2530	66.9	Negative	–	383.13785	5.6	< 1.64E‐15	[M‐H]^−^	383.13562	2.1	C_19_H_28_O_4_S_2_	MSI3	Unkwown
n_0378	65.0	Negative	–	197.04552	3.9	< 1.64E‐15	[M‐H]^−^	198.05280	0.3	C_9_H_10_O_5_	MSI3	3‐(3,4‐Dihydroxyphenyl)lactic acid
p_1078	64.4	Positive	–	209.15278	5.0	1.20E‐05	[M+H]^+^	208.14630	4.1	C_13_H_20_O_2_	MSI3	4‐Hydroxy β‐ionone
p_2652	64.0	Negative	–	365.10471	1.3	< 1.64E‐15	[M+Na]^+^	342.11622	1.6	C_12_H_22_O_11_	MSI2	Trehalose
p_3452	63.6	Negative	M1: 641.16797	642.17144	6.4	< 1.64E‐15	[M+H]^+^	640.16390	5.0	C_28_H_32_O_17_	MSI3	Quercetagetin 7‐methyl ether 3‐neohesperidoside
n_1201	62.5	Negative	–	288.06021	1.2	1.14E‐11	[M‐H]^−^	289.06740	1.2	C_17_H_11_N_3_S	MSI4	2‐[5‐(Pyrimidin‐4‐yl)thiophen‐2‐yl]quinoline
n_0421	62.5	Negative	–	204.08774	1.3	< 1.64E‐15	[M‐H]^−^	205.09500	0.2	C_8_H_15_NO_5_	MSI3	*N*‐Acetyl‐d‐fucosamine
n_4973	62.5	Negative	M: 719.16089	720.16461	7.6	< 1.64E‐15	[M‐H]^−^	720.16900	1.2	C_36_H_32_O_16_	MSI2	Sagerinic acid
n_4127	62.2	Negative	–	515.21332	7.7	2.34E‐03	[M‐H]^−^	516.21340	0.5	C_24_H_36_O_12_	MSI3	CucurbitosideF
p_1908	61.4	Negative	Fragment of 449.10553	287.05382	4.0	1.35E‐12	[M+H]^+^	449.10501	6.2	C_21_H_20_O_11_	MSI2	Luteolin‐4′‐*O*‐glucoside
n_4969	61.3	Negative	–	717.14574	7.7	< 1.64E‐15	[M‐H]^−^	718.15340	0.2	C_36_H_30_O_16_	MSI3	Salvianolic acid L
p_2660	60.8	Negative	Fragment of 394.20529	366.17393	7.2	< 1.64E‐15	[M+H]^+^	393.19400	7.3	C_24_H_27_NO_4_	MSI3	Tylophorine
p_0576	60.5	Negative	–	160.09622	1.3	< 1.64E‐15	[M+H]^+^	159.08950	6.2	C_7_H_13_O_3_N	MSI3	*trans*‐4‐Hydroxy‐l‐proline betaine
p_1252	60.2	Negative	–	227.12687	5.1	3.95E‐13	[M+H]^+^	226.12050	4.6	C_12_H_18_O_4_	MSI3	12‐Hydroxyjasmonic acid

ND, not determined.

**Fig. 2:**
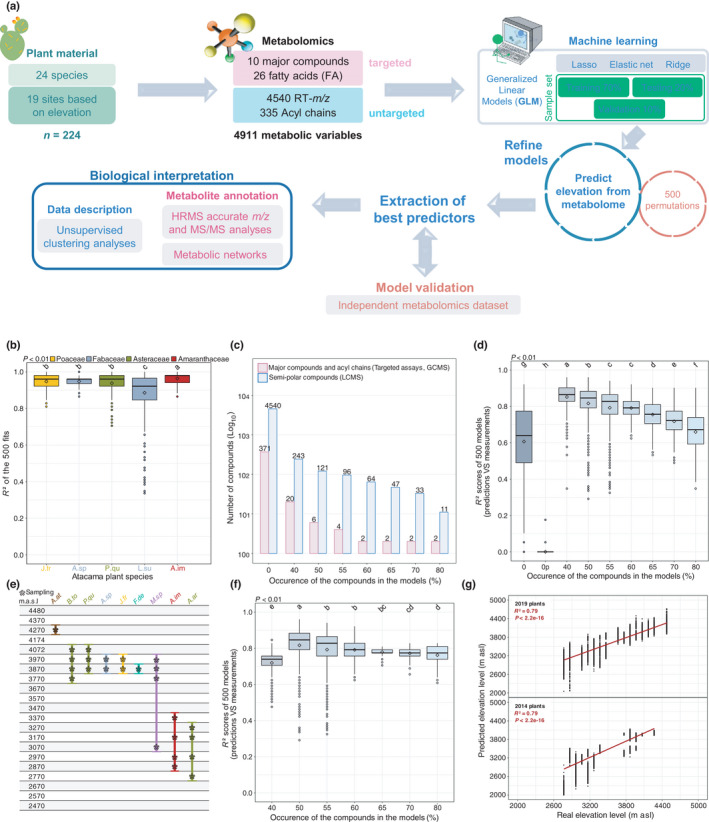
Predictive metabolomics of Atacama plants. (a) A simplified scheme of the predictive metabolomics approach used in this study. (b) Species‐specific level: *R* ^2^ scores of the fit between calculated and real elevation levels with letters indicating statistical significance (Tukey's test, *P* < 0.01). Theoretical elevations were calculated from the plant metabolome. (c) Global level: threshold of the variable occurrence defined by 500 models performed on all variables for all species. The 13 variables used in 80% represent the most relevant compounds for predicting elevation. (d) *R* ^2^ scores depending on the variable occurrence threshold (Tukey's test, *P* < 0.01). (e) Biological validation using an independent sample set from 2014. (f) *R* ^2^ scores obtained by predicting the elevation level from plants from 2014 using the multilinear equation calculated based on plants from 2019 depending on the variable occurrence threshold (Tukey's test, *P* < 0.01). (g) Predicted elevations from 2019 and 2014 plants using the best 66 markers (Pearson correlation). Compounds in (c, d, f) refer to metabolic variables *stricto sensu* before annotation.

Finally, statistical validation was complemented with biological confirmation to validate the predictive capacity of the metabolic markers using an independent sample set harvested in 2014 (Eshel *et al*., 2021) as for 2019. Using the equation calculated based on the entire 2019 dataset, a validation model predicted the elevation for each plant from the 2014 dataset. The quality of the prediction was evaluated by both the coefficient of determination and the *P*‐value observed when comparing real and predicted altitudes (Fig. 2). The same permutation protocol was used to test the likelihood of spurious prediction of 2014 validation models.

### Multivariate statistical analyses

The normalized dataset was processed through multivariate analysis such as principal components analysis (PCA) via the factominer package (Lê *et al*., 2008) in R (v.3.6.1) and two‐way orthogonal partial least square (O2PLS) via simca 16.0.1 (Umetrics, Umeå, Sweden). Tukey's tests were performed to compare the expression of the metabolic markers between species or between environments using the agricolae package (Mendiburu, 2020) with a threshold of significance established at *P* < 0.01. Finally, box plots, scatter plots, correlation plots and heatmaps were realized using the ggplot2, ggpubr, hmisc and pheatmap packages (Wickham, 2016; Kolde, 2019; Harrell Jr, 2020; Kassambara, 2020) (Pearson correlation, Ward algorithm) in *R*, respectively.

### Annotation

The best metabolic predictors were annotated using two different methods. First, MS spectra were used to analyse the isotopic patterns (^13^C, ^18^O, ^15^N and ^34^S) and speculate on the ion composition. In addition, all putative chemical formulas were calculated by the FreeStyle function within the Xcalibur 4.2 software with the following minimal and maximal constraints on chemical elements: ^14^N: 0–60, ^16^O: 0–6, ^12^C: 0–100, ^1^H: 0–200, ^32^S: 0–60, ^35^Cl: 0–60 and ^31^P: 0–60, and a mass tolerance at 10 ppm. Thus, the best candidates were chosen based on the MS analysis and screened on chemical databases (Chebi (de Matos *et al*., 2010), Metlin (Xue *et al*., 2020), DNP (http://dnp.chemnetbase.com) and Knapsack (Shinbo *et al*., 2006)). In parallel, accurate *m*/*z* values of the most discriminant monoisotopic ions were screened using the Metlin database (Smith *et al*., 2005) for putative annotation. The resulting outputs from the two methods were compared to select the best putative annotation for each ion (Table 1). In addition, MS/MS spectra of all samples were used to improve the annotation level by comparing experimental fragments with experimental MS/MS spectra available in multiple libraries such as MassBank (Horai *et al*., 2010), MzCloud (https://www.mzcloud.org) and Metlin (Xue *et al*., 2020). The annotation level of each predictor was therefore attributed following the metabolomics standards initiative confidence level (MSI levels) (Sumner *et al*., 2014).

### Pathway analysis and metabolic networks

The 39 predictors were screened through chemical databases and integrated into a metabolic network to better interpret their role in the plant's response to extreme conditions. The KEGG identifiers were determined using the KEGG database (Kanehisa *et al*., 2014) on the 39 molecules if available or on chemically related compounds (Table S3). Thereafter, a pathway enrichment analysis was realized using the MetaboAnalyst (Xia *et al*., 2015) and PlantReactome (Naithani *et al*., 2019) databases, and combined with the best markers integration into a preexisting *Arabidopsis thaliana* metabolic network via MetExplore (Cottret *et al*., 2010).

## Results

### Plant diversity in the extreme conditions of the Atacama Desert

The Atacama Desert represents one of the harshest environments for plant life (Jordan & Kirk‐Lawlor, 2014; Díaz *et al*., 2016), where plants must endure the major abiotic stresses currently threatening agriculture. The Talabre–Lejía Transect (TLT) spans an elevation gradient covering three different plant communities: the poorly vegetated Prepuna (2400–3300 m asl), the Puna shrubland (3300–4000 m asl) and the high Andean Steppe (4000–4500 m asl) (Fig. 1a) (Díaz *et al*., 2016; Eshel *et al*., 2021). Water availability increases and temperature decreases with altitude, while high solar irradiance and very low nitrogen levels are critical constraints throughout the TLT (Eshel *et al*., 2021). Rainfall ranges from 20 mm yr^−1^ in the prepuna to 160 mm yr^−1^ in the steppe, illustrating the extreme aridity as compared to other plant‐sheltering deserts (Báez & Collins, 2008; Li *et al*., 2015; Díaz *et al*., 2016; Ziaco *et al*., 2018). The daily average solar irradiance of 600 W m^−2^ d^−1^ along this transect is three times higher than many deserts and high mountain ecosystems (Bo *et al*., 2009; Zhang *et al*., 2010; Arancibia‐Bulnes *et al*., 2014). In addition, low total nitrogen (average 9 mg kg^−1^) throughout the transect, low phosphorus levels (6–20 mg kg^−1^) and high salinity in Prepuna sites add to the harsh conditions plants must endure. Nonetheless, plant life in this ecosystem of the Atacama can be traced back to 45 000 yr ago (Latorre *et al*., 2002; Díaz *et al*., 2019) and has thrived in such extreme conditions since probably 12 million years ago (Jordan & Kirk‐Lawlor, 2014). Hence, this ecosystem represents a unique resource of adaptive mechanisms potentially relevant to engineer crop resilience. Interestingly, deep‐sequencing of 32 dominant species representing the major clades highlighted common and specific strategies relevant for plant survival (Eshel *et al*., 2021). In this context, we collected 21 of these 32 plant species based on their coverage in their natural ecosystem. We complemented this set with one Cactaceae, one Solanaceae and one Boraginaceae to finally represent relevant biodiversity covering annual and perennial plants, different carbon fixation systems (i.e. C_3_, C_4_ and CAM) and different lifespans such as shrubs and herbs (Fig. 1c; Table S1). Clear distinctions regarding the distribution of life‐form and carbon fixation types have been highlighted where all annuals and C_4_ plants were observed at an elevation of < 3870 m asl. Additionally, while some species were relatively specific to a single environment (e.g. *Moschopsis monocephala*), others had a wide distribution along the transect area that we divided into 19 sites (each 100 m asl) (Fig. 1b). We also selected 11 agronomic and ornamental species based on their plant family to analyse and compare using the same experimental procedures (as explained in the ‘Materials and Methods’ section). A taxonomic analysis performed on the Atacama and agronomic plant species via the NCBI taxonomy browser revealed the relationships between the 14 Atacama plant families (Fig. 1d). Interestingly, this sample set of 23 angiosperms and one gymnosperm included well‐known resilient plant families such as Cactaceae and Boraginaceae (Ma *et al*., 2010) together with species of economic interest such as those in Poaceae, Asteraceae, Fabaceae and Solanaceae. In addition, the 11 agronomic and ornamental plant species covered five of the 14 Atacama plant families (including the most widespread ones in Poaceae, Fabaceae, Asteraceae and Solanaceae).

### Predictive metabolomics reveals a core metabolic set in multiple resilient species

To gain insight into the mechanisms by which these extremophile plants adapt to the extreme conditions of the Atacama Desert and respond to environmental variations, we performed multiplatform metabolomics to screen both primary and secondary metabolisms from the aerial tissues of Atacama plants and agronomic species (Fig. 2a). Quantitative evaluation of 10 major compounds by biochemical phenotyping (used as key physiological indicators) and 26 fatty acids by GCFID highlighted a significant reduction in Chl, nitrate and protein content in Atacama species when compared to 11 known crops and ornamental plants (Fig. S2). In addition, the unknown biochemical diversity of these extreme Atacama plants was analysed through untargeted metabolomics using GCMS and LCMS, which resulted in 335 acyl chains and 4540 semipolar features after preprocessing (Fig. S2). Given that the phytochemical diversity fluctuated with environmental conditions along the elevation gradient (400–2000 m asl) (Defossez *et al*., 2021), GLM was used to test whether the metabolome (4911 variables) could predict environmental conditions (Fig. 2a). Elevation represents the integration of abiotic factors (Carpenter, 2005), among which climatic and edaphic factors have been previously described (Eshel *et al*., 2021) (Fig. 1b). Thus, the elevation level of the 19 sampling spots was used as a proxy of the 19 environmental conditions analysed. First, the possibility of calculating the elevation levels from five different plant species selected based on both their biomass and coverage along the elevation gradient was evaluated. For each species, 80% of the sample set (i.e. training sets) was used for the regression analysis. The equation was then used to calculate elevation for the 20% of the sample set remaining (i.e. testing set). Interestingly, the resulting average *R* ^2^ from 500 models (i.e. fits between calculated and measured elevation) ranged between 0.88 and 0.96 depending on the species (Fig. 2b). These results indicate the plant metabolome integrates environmental variations. Environmental conditions elicited characteristic metabolic patterns in which compounds correlate with elevation, allowing us to infer the altitude from which the sample was collected.

Moreover, estimating the altitude (i.e. resulting as an environment proxy) from metabolic data alone for species from several plant families raised the question of whether generic mechanisms serve as a basis for adaptation to extreme environments such as the Atacama Desert. To address this question, we used GLM on the entire dataset divided into a training set (70%), a testing set (20%) and a validation set (10%) since the total size of the dataset was sufficient (*n* = 224). We thus predicted the plant environment (i.e. elevation level) and highlighted the shared metabolic predictors (Fig. 2a). A first modelling step determined the predictive capacity of the 4911 metabolic variables, represented by their percentage of use in the models. Consequently, a threshold of 40% (i.e. variables used in more than 40% of the 500 models) included 263 features while 80% involved the best 13 metabolic predictors (Fig. 2c). Subsequently, each threshold was processed to exclude the nonpredictive features and tightly select the best ratio between the predictive capacity and the number of metabolic variables. The plant environment was considerably predictable at 66% and 79% using 13 or 66 markers, respectively (Fig. 2d). Lower thresholds (e.g. 40%) allowed better predictions but yielded less robust predictors (i.e. higher SD). Importantly, 500 permutation sets involving randomly assigned elevation levels were developed to test the likelihood of spurious predictions, which led to a mean *R* ^2^ of 0% and thus statistically validated the GLM‐based modelling approach. Hence, we demonstrated that common features could greatly predict plant environments (79%), independently of the species and family.

To further test the robustness of such predictions, we biologically confirmed the predictive capacity of the metabolic features using an independent dataset composed of nine Atacama plant species harvested in 2014 and covering 12 environments (2770–4270 m asl) (Fig. 2e). The linear equation developed using the 2019 samples was then applied to the 2014 dataset to estimate elevation levels, thereby resulting in similar predictive patterns between 2019 and 2014 (Fig. 2f). Altogether, both mean *R* ^2^ prediction and SD results pointed towards an ideal threshold of 60% (66 variables), which allowed a prediction at 79% for both years (*P* < 2.2e^−16^) (Fig. 2g). These results hence confirm that plants harbour a core set of metabolites to adapt to the environmental constraints.

### A not‐so‐specialized set of secondary metabolites also detected in agronomic and ornamental plant species

**Fig. 3:**
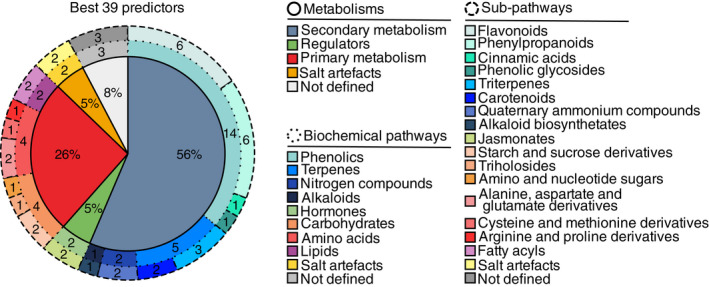
Pathway analysis of the 39 markers. Metabolism, biochemical pathways and subpathways were elucidated by screening the KEGG identifiers through the MetaboAnalyst, PlantReactome and MetExplore databases.

Next, we annotated the best 66 predictors using both accurate *m*/*z* values and MS/MS analysis. This annotation process allowed us to exclude the fragments observed among the 66 features (Table S4), finally retaining 39 metabolic predictors without remarkable impact on the average *R* ^2^ (Fig. S2a). The MSI annotation level for each predictor is presented in Table 1. Notably, the best predictor was starch, while 37 metabolites were referred to semipolar compounds (Table 1). Only six markers were positively correlated to elevation, while the intensity of the remaining compounds decreased with elevation (Fig. S3b). Remarkably, predictors in Atacama plant species were also found in several agronomic and ornamental plants (Tables S5, S6), demonstrating the ubiquitous nature of these metabolites. These 39 compounds were queried in biochemical databases (e.g. Kegg, PlantReactome) to perform a pathway analysis (Table S3) and placed into a preexisting *A. thaliana* metabolic network available on MetExplore (Kanehisa *et al*., 2014) (Fig. S4). More than half of the markers were involved in secondary metabolism (56%), while primary metabolism and regulators (e.g. jasmonates) covered 31% in total (Fig. 3). The remaining 13% included three unknown compounds and two salt artefacts that combined sodium and magnesium to formic acid, suggesting salt hyperaccumulation processes (Figs 3, S3a). Notably, starch, trehalose and amino acid‐related pathways were involved in crosstalk with the biosynthesis of secondary metabolites, while the central place of raffinose was highlighted in galactose metabolism involving other oligosaccharides known for their role in abiotic stress tolerance (Vinson *et al*., 2020) (Fig. S4a). In addition, phenolics represented the major enrichment observed in Atacama plant species with 14 of the 39 markers. While alkaloids and N‐containing compounds (e.g. proline betaine, or polyamines combined with flavonoids) were included in the best markers, flavonoid, phenylpropanoid and terpenoid pathways were clearly overrepresented (Figs 3, S4). Last but not least, despite their classification into primary or secondary metabolisms, a relevant proportion of these 39 markers also referred to redox homeostasis based on their chemical nature or interactions with ascorbate or glutathione pathways (Fig. S4), suggesting the importance of redox homeostasis in the adaptation to hostile environments. Overall, predictive metabolomics reveals that plant metabolism greatly reflects environmental fluctuations in extreme ecosystems, as also pinpointed by a core set of metabolites (involved in secondary, primary and redox pathways) capable of predicting at 79% the plant environment independently of the plant species. These findings thus confirm a central place for generic metabolic pathways underpinning plant adaptation to environmental constraints.

### The plant metabolome is tailored to environmental constraints

Elevation integrates a wide range of abiotic factors, among which edaphic variables were measured in each of the 19 sampling spots. Climatic variables such as temperature, soil water content (SWC, representing the interaction between precipitation and soil properties), precipitation and solar irradiance were measured via two stations (at 3060 and 4090 m asl). Theoretical values of these factors along the elevation gradient for the 19 environments were predicted considering a linear distribution that was confirmed by field measurements (Fig. S5). Elevation correlated closely with most environmental parameters in the Atacama Desert (Fig. S6). Thus, an analysis combining PCA and O2PLS was performed to unravel the elevation factor and highlight the relationship between the 39 best predictors and environmental factors.

**Fig. 4:**
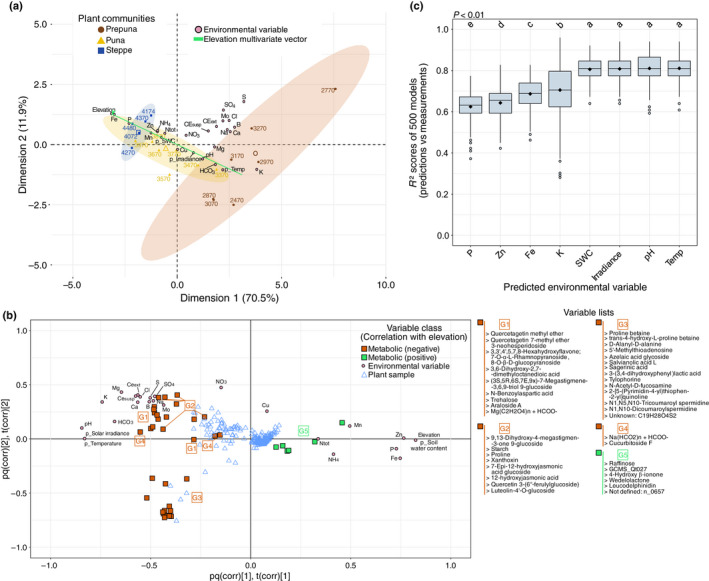
Decomposition of the elevation factor and environment–metabolome covariation. (a) Principal component analysis biplot. Discrimination of the sampling spots by the environmental data. SWC represents the soil water content while p_ represents a partially predicted parameter. (b) Two‐way orthogonal partial least squares analysis describing the covariation between environmental and metabolic data. Hierarchical clustering analysis was realized with Pearson correlation and Ward algorithm. (c) Boxplot showing the average *R* ^2^ scores (500 models) performed on the best discriminant environmental variables using the 66 best metabolic markers. Letters indicate statistical significance (Tukey's test, *P* < 0.01). SWC, soil water content; Temp, temperature.

First, PCA was used to reveal the influence of elevation on the climatic and edaphic conditions. The first two components of the PCA model explained 82.4% of the total variance of the dataset (Fig. 4a) and showed clear discrimination of the plant communities (i.e. Prepuna, Puna and Steppe) along a multivariate vector that represented the elevation gradient. Also, a second plane defined by several minerals divided the different environments belonging to the Prepuna ecosystem, which did not occur with Steppe spots. Hence, the previously predicted elevation factor was here depicted by a multivariate vector represented mostly by edaphic variables (e.g. pH, P, K), temperature and solar irradiance.

Second, we predicted the covariation between environmental factors and the best 39 markers using an O2PLS analysis (Fig. 4b). Remarkably, 95% of the variation observed in the environmental dataset was covered by the metabolic features (Table S7). Congruently with the correlation matrix and PCA (Fig. 4), the best predictors were distributed primarily along the first component representing elevation and, to a lesser extent, linked to several edaphic factors such as sulphur. The O2PLS biplot further highlighted a remarkable separation between metabolic compounds positively or negatively correlated with elevation along the first plane. In particular, five phytochemicals determined by Pearson clustering and including raffinose were plotted with regard to temperature. These results indicate that the discriminant capacity of elevation resulted primarily from temperature, solar irradiance, SWC and several edaphic factors. These PLS predictions were also confirmed by GLM, where the best predictions using the 39 best markers on independent environmental parameters were obtained for SWC, solar irradiance, pH and temperature (Fig. 4c).

Overall, we provide a valuable approach combining metabolomics, GLM and multivariate statistical analyses. Exploiting multiple species in a natural environment can successively reveal generic mechanisms of interest and disentangle complex systems into specific environmental parameters (i.e. climatic and edaphic).

## Discussion

### Predictive metabolomics demonstrates a generic metabolic toolbox for plant adaptation to extreme habitats

Ecological metabolomics, which allowed study of the interaction between plant metabolism and environment, has attracted scientific curiosity for over 50 yr (Sardans *et al*., 2020). While studies on single plant species led to limited results when transferred to crops, a metaanalysis of individual studies highlighted metabolic convergences in plant species inhabiting extreme environments (Dussarrat *et al*., 2021), encouraging plant researchers to move towards a more holistic approach. Strikingly, our approach combining ecological metabolomics with GLM‐based modelling was able to predict the plant environment with an *R* ^2^ as high as 0.96 within given species (Fig. 2b) and 0.79 between species (Fig. 2). Such values are far above correlation coefficients usually obtained with phenotypic traits (Laughlin *et al*., 2012; Poorter *et al*., 2019), which are also more difficult to score (Laughlin & Messier, 2015), making metabolic markers ideal soft traits.

All Atacama plant species harboured low Chl levels as compared to agronomic species, which could result from an adaptive response to high solar irradiance or the meagre availability of other resources such as water or nitrogen (Hikosaka *et al*., 2003), as further illustrated by the very low nitrate and protein contents (Fig. S2). Our results suggest that these 24 species, belonging to 14 families, also use a common metabolic toolbox, underpinned by the 39 metabolic markers revealed by our modelling approach, to cope with their environment (Fig. 2g). This toolbox is certainly generic as the same metabolic traits were found in several agronomic and ornamental families, validating the ubiquitous nature of this core set. Main differences were observed for flavonoid‐ and terpenoid‐related predictors, which accumulated to high levels in Atacama plants (Fig. S7). In addition, levels of raffinose and 4‐hydroxy β‐ionone (a compound related to carotenoid degradation) were higher in Steppe species than most temperate species, while Prepuna species accumulated proline derivative compounds. Several markers such as quercetin glucoside and coumaroyl‐spermidine relatives were only detectable using a lower threshold in agronomic families (Table S6). By contrast, several hormone and primary metabolism‐related predictors (e.g. jasmonates, trehalose) did not present major changes between extreme and nonadapted species, except for Chls and proteins, which were lower in Atacama plants. Overall, these observations question the potential adaptive capacity of these agronomic and ornamental species, which naturally develop in mainly temperate regions. The possibility that the genome of agronomic plants would already permit the synthesis of metabolites relevant for plant survival in harsh lands is supported by the presence of *Solanum chilense* (closely related to the cultivated *S*. *lycopersicum*) in the 24 Atacama species studied. From an evolutionary point of view, this further suggests that it is easier to modify the regulation of existing metabolic pathways than to create new ones. Also, the high *R* ^2^ score prompts the question of how species‐specific metabolic adaptations could provide a selective advantage. Hence, the relationship between these metabolic markers and the genetic adaptations discovered in Atacama plants (Eshel *et al*., 2021) deserves further investigation. In particular, several species colonized a wide elevation gradient, which suggests high plasticity (Fig. 1). Consequently, metabolic adjustments enabling the acclimation or adaptation to extreme conditions are not necessarily the result of a long evolutionary process.

### Involvement of the best metabolic predictors in extreme environment adaptation

The influence of the elevation gradient on metabolic patterns is mainly reflected in a multivariate vector involving temperature, SWC, irradiance and pH (Fig. 4a). However, extremophiles also face other environmental pressures such as the mineral imbalance observed in the Atacama Desert that deserves closer examination (Fig. 1b). Thus, the success of thriving in the Atacama Desert elevation gradient would result from the ability to cope with daily subzero temperatures at the top of the transect and hyperaridity and high salinity at the bottom, or to manage the balance between carbon input and access to other critical resources such as water.

Starch was the best predictor (Table 1), while trehalose and raffinose were among the top predictors, confirming a suitable place for carbohydrates in the resilience mechanism (Fig. S4). In the Atacama Desert, solar irradiance is not a limiting factor for carbon entry, and even threatens plant survival (Eshel *et al*., 2021). The shallow protein level observed in all Atacama plant species (Fig. S2) suggests that plant growth is very low (Elser *et al*., 2008). Therefore, carbon that does not fuel plant growth and protein turnover could be transiently stored as starch or allocated to protective systems against oxidative stress induced by other environmental factors such as water availability, temperature and salinity. Starch, metabolism of which is known to play a major role against abiotic stress (Thalmann & Santelia, 2017), can be used as a carbon source for the synthesis of protective compounds when environmental conditions become harsher, while its accumulation could be linked to sodium scavenging in halophytes (Thalmann & Santelia, 2017), for instance. The strong negative correlation of starch with elevation would result from a trade‐off with the production of osmolytes and other protective compounds required at the highest elevations, where daily subzero temperatures occur. Alternatively, the lower efficiency of transitory starch remobilization under low temperature could explain low starch contents at high levels. Conversely, raffinose negatively correlated with temperature, validating the central place of raffinose family oligosaccharides in cold tolerance (Vinson *et al*., 2020). However, several predictors were fatty acyls within the primary metabolism and jasmonates, which supported a role of lipid remodelling in extreme environments (Cao *et al*., 2016; Dussarrat *et al*., 2021).

More than half of the 39 best markers were involved in secondary metabolism (Fig. 3). Remarkably, phenolics (14/39 compounds) were increased at lower elevations, which would help plants cope with both the very low water availability and high salinity of these lands. This protective process has already been described in multiple extremophile plant species (Dussarrat *et al*., 2021). Phenolic antioxidant properties, mainly for cinnamic acid and quercetin derivatives (extensively represented in the best predictors), enhance photoprotection and resilience to abiotic stresses (Agati & Tattini, 2010). Regarding terpenoids, the presence of xanthoxin and 4‐hydroxy β‐ionone (a carotenoid degradation product) within the 39 best markers supported the role of carotenoids per se as well as their degradation in extreme climate resilience (Table 1). Despite their well‐described antioxidant role, Havaux (2014) discussed the link between the catabolism of carotenoids and plant defence, as their cleavage leads to hormonal compounds (e.g. abscisic acid) or redox signalling. Also, the accumulation of N‐related compounds could be attributed to their role in osmoregulation (e.g. proline betaine). The contribution of phenolics that conjugate polyamines and other molecules (e.g. tricoumaroyl spermidine) is more complex, despite a growing body of evidence for their implication in stress mitigation (Pál *et al*., 2018). Most importantly, our study linked plant survival under harsh conditions to redox metabolism since the majority of metabolic markers directly or indirectly involve redox homeostasis. This was exemplified by primary compounds of the glutathione and ascorbate pathways, metabolites for ROS processing including carotenoids, as well as potential links between the biosynthesis of several compounds and NAD metabolism (Fig. S4), all participating in oxidative stress signalling (Decros *et al*., 2019; Dussarrat *et al*., 2021). Among the best predictors is proline, accumulation of which in response to osmotic stress has been widely documented and recently attributed to redox homeostasis (Szabados & Savouré, 2010). Alternatively, accumulated levels of amino acids could serve as metabolic intermediates for the synthesis of more complex secondary metabolites with stress‐responsive functions.

Overall, uncovering the metabolic characteristics of Atacama species highlighted the linear encapsulation of environmental fluctuations by the plant metabolome (involving primary, secondary and redox pathways), and the use of generic metabolic mechanisms to adapt to extreme growth conditions. Such an approach (multispecies harvested in extreme environments) offers promising perspectives in both ecological chemistry and stress physiology worldwide. A fascinating perspective will be to research the genetic and molecular mechanisms that control the levels of these metabolic markers.

## Author contributions

TD, YG, DR, RG and PP designed and planned the project. TD, CL, FD and RG performed the fieldwork. TD, SP, PP, SB, AF, CC, PVD, KV and JJ conducted metabolic or bioinformatic experiments and analyses. DJ uploaded all data online. TD, YG, DR, RG and PP integrated and analysed the results of all the experiments. TD, RG and PP wrote the paper with feedback form all the coauthors.

## Supporting information


**Fig. S1** Correlation between starch content and sampling time. Absence of correlation between the time of sampling and starch content in plant samples.
**Fig. S2** Changes in major compounds in Atacama plants. (a) Depiction of the biochemical diversity observed in Atacama plants and 11 agronomic or ornamental species.
**Fig. S3** Best metabolomics predictors in Atacama plants. (a) Predictive capacity and clustering of the best metabolic markers.
**Fig. S4** Metabolic networks. Best markers were mapped into a preexisting *A. thaliana* metabolic network using MetExplore.
**Fig. S5** Validation of the environmental prediction. Linearity of environmental parameters with elevation.
**Fig. S6** Decomposition of the elevation parameter. Correlation plot of the environmental data.
**Fig. S7** Best metabolic predictors in agronomic and ornamental plant species. Clustering analysis of the best metabolic predictors between Atacama and agronomic and ornamental plant species.

**Table S1** (a) Sample data from Atacama plants. (b) Sample data from agronomic and ornamental plants.
**Table S2** Metabolic dataset.
**Table S3** Pathway analysis.
**Table S4** Annotation table of markers.
**Table S5** Markers in control plant. Intensity of the best metabolic markers in agronomic and ornamental plants. The detection limits were fixed to 25 000 or 10 000.
**Table S6** Markers threshold 100. Intensity of the metabolic markers that could not be observed with a 10k noise threshold. Those markers were manually checked on one sample of each nonextreme species and one Atacama species with a threshold of 100.
**Table S7** O2PLS model.
Please note: Wiley Blackwell are not responsible for the content or functionality of any Supporting Information supplied by the authors. Any queries (other than missing material) should be directed to the *New Phytologist* Central Office.

## Data Availability

The metabolic dataset and all metadata were deposited online using Dataverse INRAE (https://dx.doi.org/10.15454/UUBXIF) and following the Findable, Accessible, Interoperable, Reusable (FAIR) principles (Jacob *et al*., 2020).
