# Supplementary material for: Predictive metabolomics of multiple Atacama plant species unveils a core set of generic metabolites for extreme climate resilience
Source: New Phytol. 2022 Apr 5;234(5):1614–28. doi: 10.1111/nph.18095 (PMC9324839; doi:10.1111/nph.18095)
Supplement: Supplementary file 1 — Fig. S1 Correlation between starch content and sampling time. Absence of correlation between the time of sampling and starch content in plant samples. Fig. S2 Changes in major compounds in Atacama plants. (a) Depiction of the biochemical diversity observed in Atacama plants and 11 agronomic or ornamental species. Fig. S3 Best metabolomics predictors in Atacama plants. (a) Predictive capacity and clustering of the best metabolic markers. Fig. S4 Metabolic networks. Best markers were mapped into a preexisting A. thaliana metabolic network using MetExplore. Fig. S5 Validation of the environmental prediction. Linearity of environmental parameters with elevation. Fig. S6 Decomposition of the elevation parameter. Correlation plot of the environmental data. Fig. S7 Best metabolic predictors in agronomic and ornamental plant species. Clustering analysis of the best metabolic predictors between Atacama and agronomic and ornamental plant species. [file NPH-234-1614-s002.pdf]

New Phytologist Supporting Information. Predictive metabolomics of multiple Atacama plant species unveils a core set of generic metabolites for extreme climate resilience.

Thomas Dussarrat, Sylvain Prigent, Claudio Latorre, Stéphane Bernillon, Amélie Flandin, Francisca P. Díaz, Cédric Cassan, Pierre Van Delft, Daniel Jacob, Kranthi Varala, Jérôme Joubes, Yves Gibon, Dominique Rolin, Rodrigo A. Gutiérrez, Pierre Pétriacq. 28 February 2022.

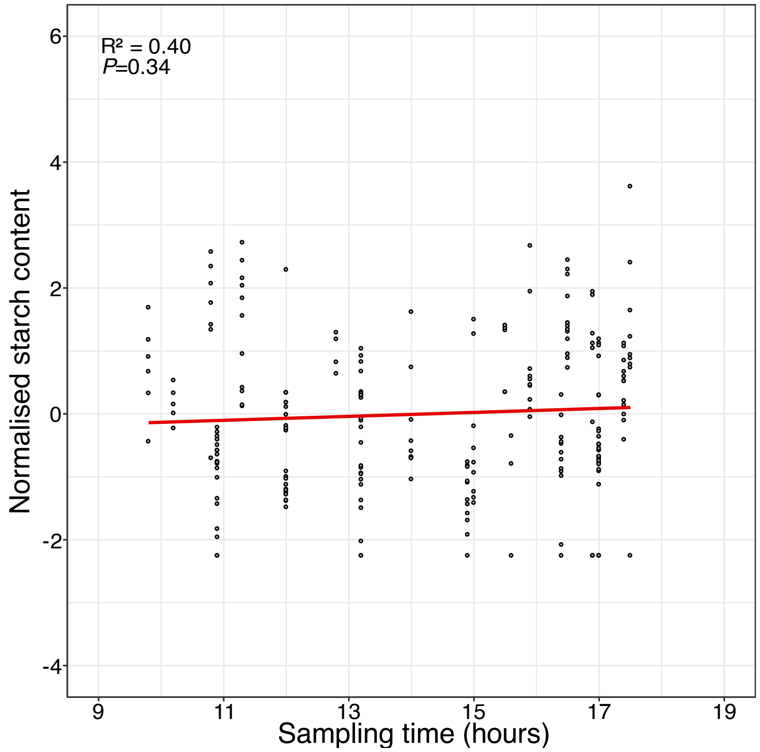

Fig. S1 | Correlation between starch content and sampling time. Absence of correlation between the time (in hours) of sampling and starch content in plant samples (Pearson correlation).

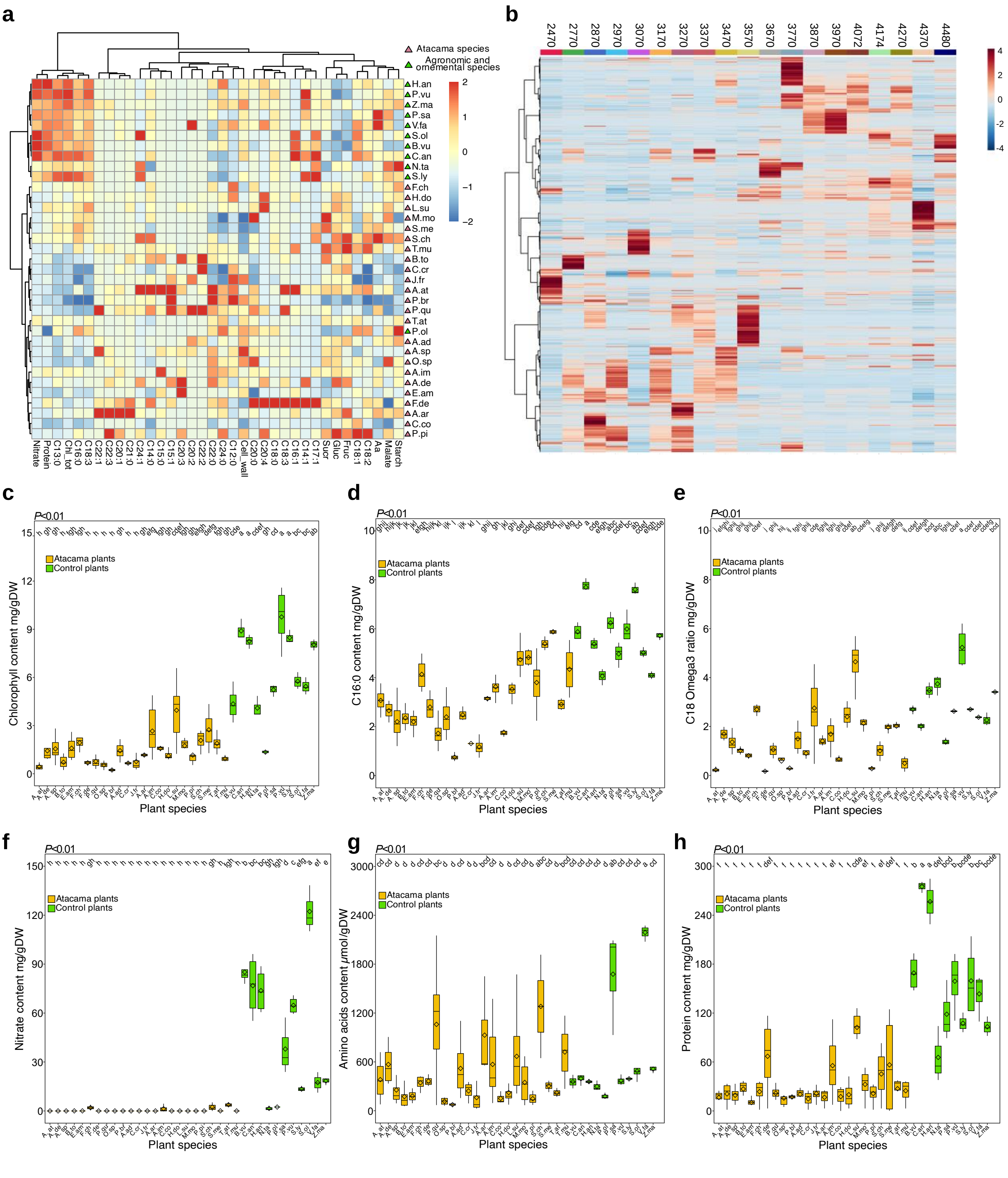

Fig. S2 | Changes in major compounds in Atacama plants. a Two-dimensional clustering analysis (Pearson correlation, Ward algorithm) showing a heatmap of major compounds detected by targeted assays and GCFID of 24 Atacama plants *versus* 11 agronomic or ornamental species. **b** Biochemical diversity observed in the 19 sites using targeted and untargeted analyses. Only significant variables were used (Tukey's test with $P<0.01$) **c**-**h** Details of the major discriminant compounds. Tukey's test were performed with $P<0.01$. The box in each box plot illustrates the lower, median and upper quartile values, and the vertical lines show the range of the concentration variation in samples while squares and circles represent the mean concentration and potential outliers, respectively.

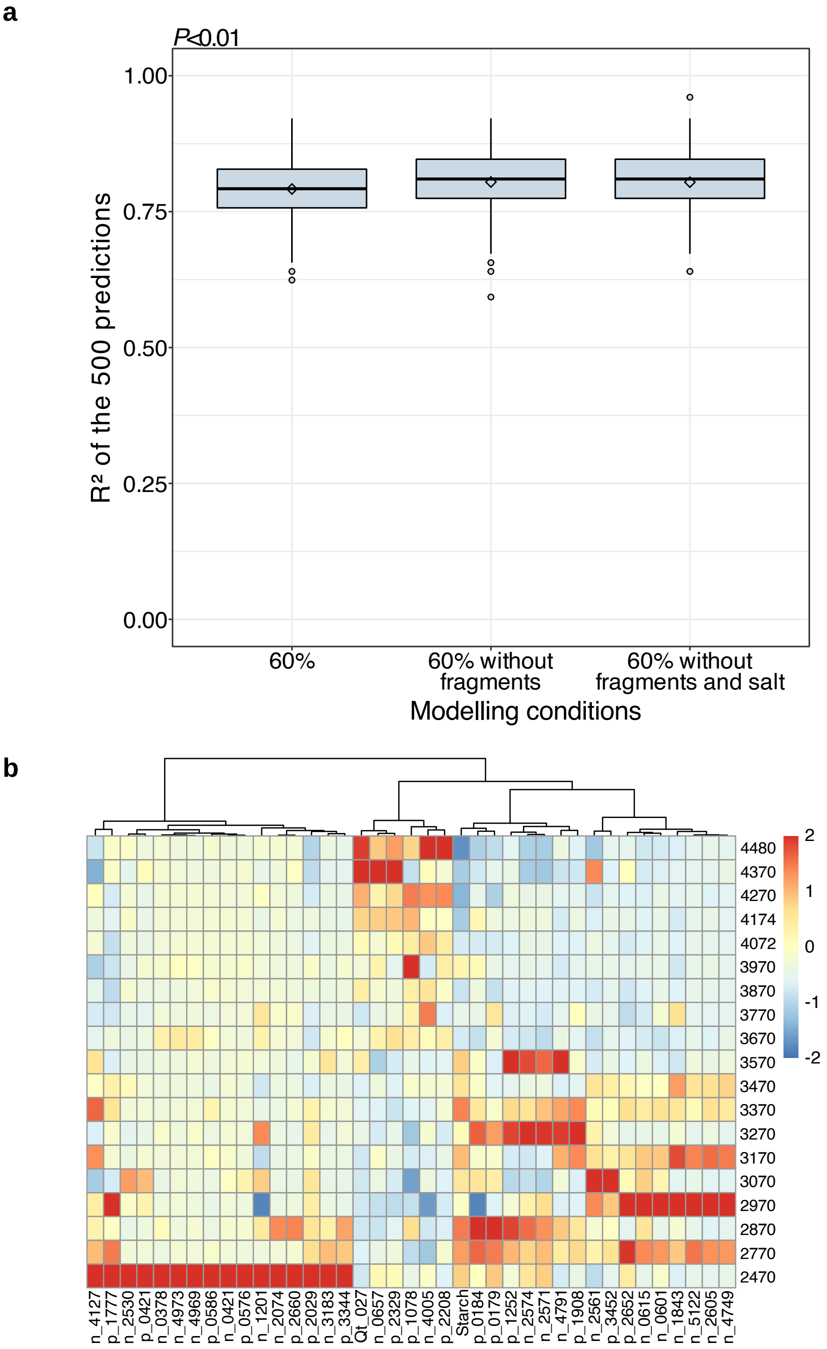

Fig. S3 | Best metabolomics predictors in Atacama plants. a Boxplot showing R^2 of the 500 models performed on the best metabolic markers (threshold 60%) with or without fragments and Formic acid + salt compounds. The box in each box plot illustrates the lower, median and upper quartile values, and the vertical lines show the range of the R^2 variation in samples while squares and circles represent the mean R^2 and potential outliers, respectively. **b** Clustering analysis (Pearson correlation, Ward algorithm) with a heatmap illustrating the relation between best metabolic predictors and elevation.

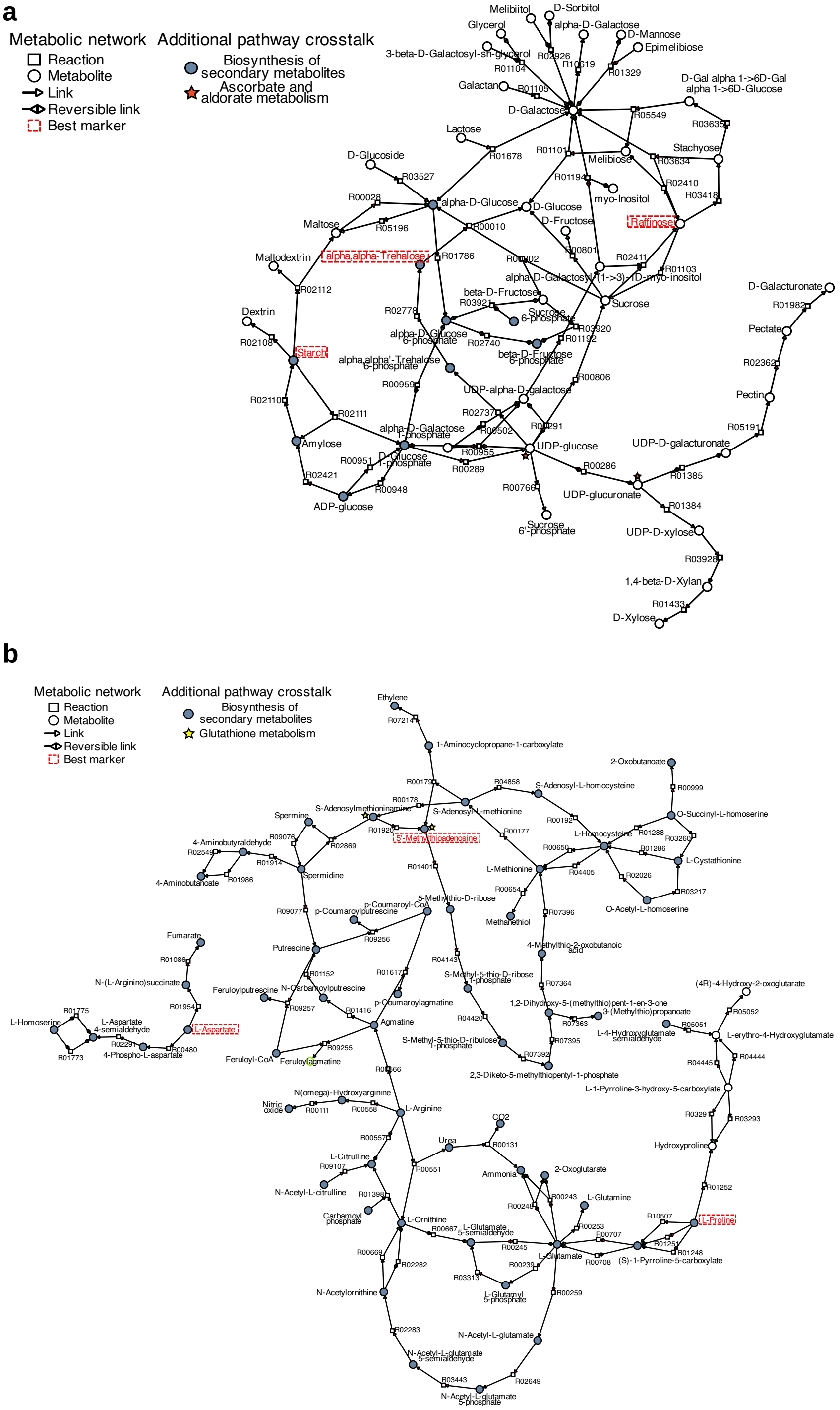

Fig. S4 | Metabolic networks. Best markers were mapped into a pre-existing *A. thaliana* metabolic network using MetExplore**. a** Crosstalk between the sub-pathways “Starch and sucrose metabolism” and “Galactose metabolism”. **b** Crosstalk between the sub-pathways “Cysteine and methionine derivatives”, “Arginine and proline derivatives” and “Alanine, aspartate and glutamate derivatives”. **c** “Flavonoid biosynthesis” sub-pathway. **d** “Carotenoid biosynthesis” sub-pathway. **e** “Phenylpropanoid biosynthesis” sub-pathway.

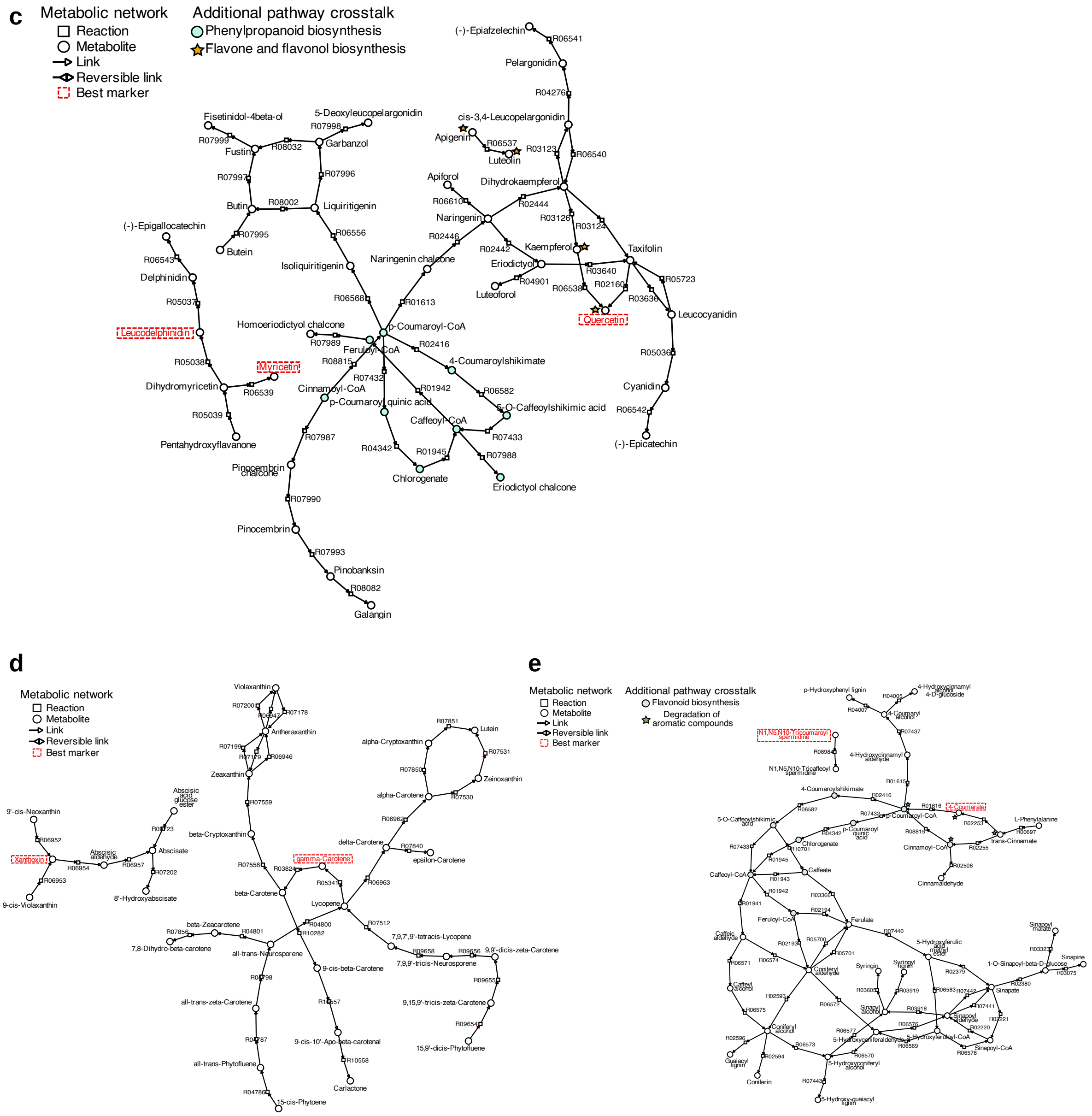

Fig. S4 | Metabolic networks. Best markers were mapped into a pre-existing *A. thaliana* metabolic network using MetExplore**. a** Crosstalk between the sub-pathways "Starch and sucrose metabolism" and "Galactose metabolism". **b** Crosstalk between the sub-pathways "Cysteine and methionine derivatives", "Arginine and proline derivatives" and "Alanine, aspartate and glutamate derivatives". **c** "Flavonoid biosynthesis" sub-pathway. **d** "Carotenoid biosynthesis" sub-pathway. **e** "Phenylpropanoid biosynthesis" sub-pathway.

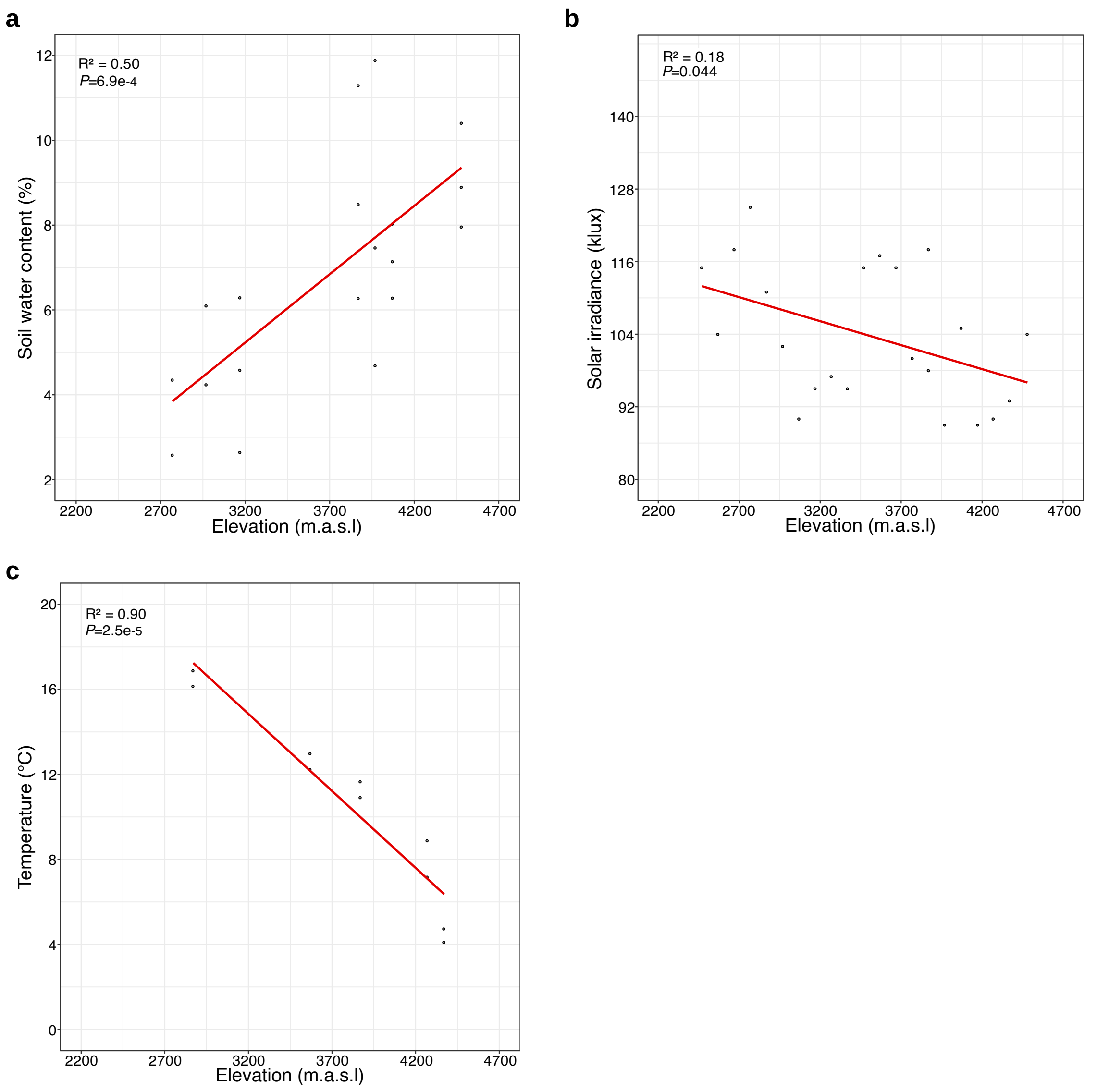

Fig. S5 | Validation of the environmental prediction. Distribution of (**a**) soil water content, (**b**) solar irradiance and (**c**) temperature along the elevation gradient (Pearson correlation, $P<0.05$). Soil samples were collected near the plants of interest. The moisture content of the soil was determined by dividing the fresh weight by the dry weight after one week of freeze drying. Solar irradiance was measured using a lux meter near the plants of interest. Temperature was measured using two thermometers.

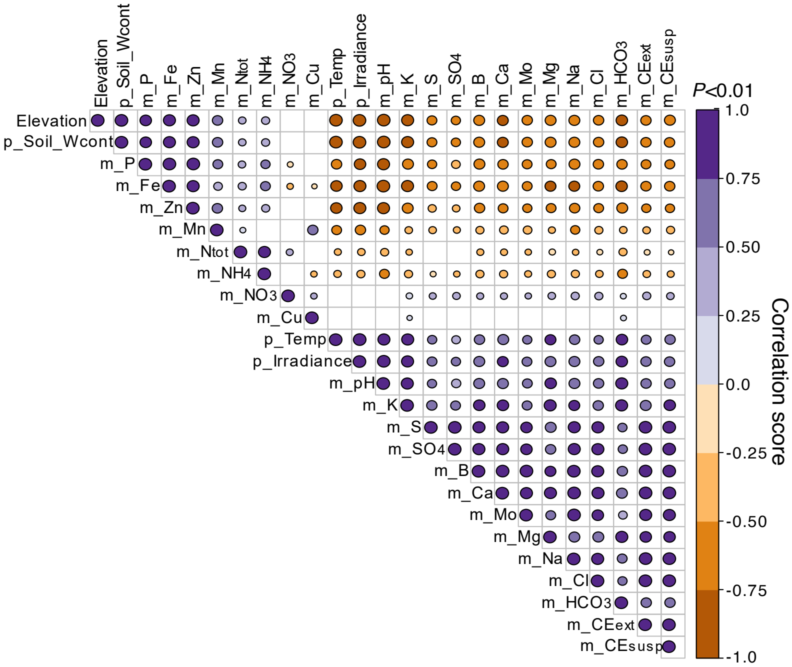

Fig. S6 | Decomposition of the elevation parameter. Correlation plot of the environmental data (*P*<0.01, Pearson correlation).

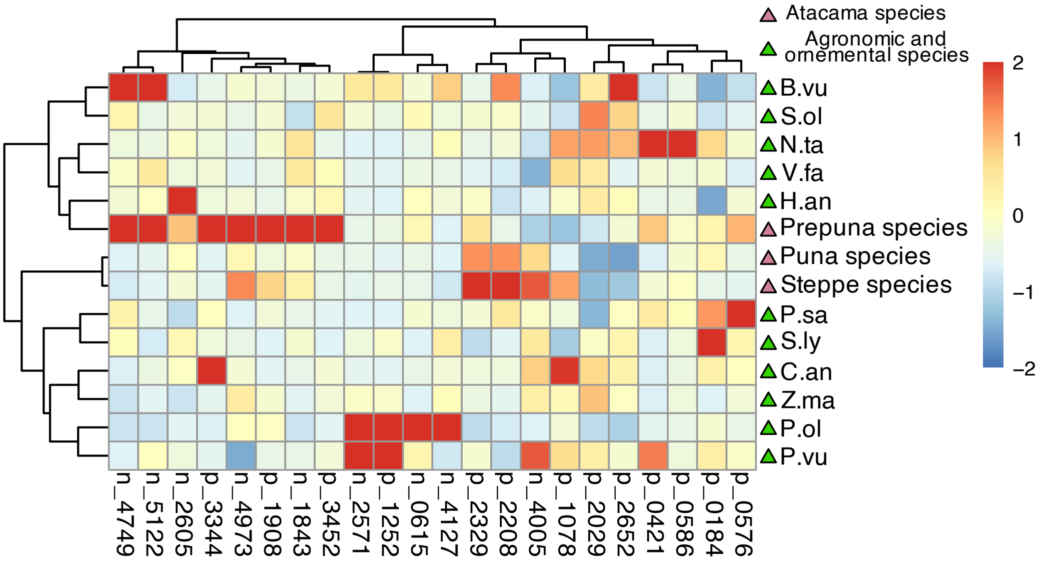

Fig. S7 | Best metabolic predictors in agronomic and ornamental plant species. Clustering analysis of the best metabolic predictors between Atacama and agronomic and ornamental plant species (Pearson correlation, Ward algorithm).
